# Hunterian Lectures on Hernia

**Published:** 1889-12

**Authors:** 


*Hunterian Lectures on Hernia.* By C. B. LOCKWOOD, F.R.C.S. Pp. 168. London: H. K. LEWIS. 1889.

Such a mass of rubbish is annually sent out in booklet-form as personal opinions and abridgments, and handbooks and so forth, that we are especially delighted to find a booklet which is anything but trash, and one wherein facts, laboriously collated, and judiciously interpreted, are made to speak for themselves. Mr. Lockwood's *Hunterian Lectures* are undoubtedly one of the most notable of recent contributions to the pathology of hernia, and must have influence on future studies.

There is here and there a little laxity in grammatical form. Throughout the work "ilium" is made to mean both bone and bowel.

